# The role of mindfulness in distress and quality of life for men with advanced prostate cancer

**DOI:** 10.1007/s11136-016-1341-3

**Published:** 2016-06-17

**Authors:** Suzanne K. Chambers, Elizabeth Foley, Samantha Clutton, Robert McDowall, Stefano Occhipinti, Martin Berry, Martin R. Stockler, Stephen J. Lepore, Mark Frydenberg, Robert A. Gardiner, Ian D. Davis, David P. Smith

**Affiliations:** 1 Menzies Health Institute Queensland, Griffith University, Gold Coast, QLD 4222 Australia; 2 Cancer Council Queensland, Brisbane, Australia; 3 Prostate Cancer Foundation of Australia, Sydney, Australia; 4 University of Queensland Centre for Clinical Research, University of Queensland, Brisbane, Australia; 5 Health and Wellness Institute, Edith Cowan University, Perth, Australia; 6 Australian and New Zealand Urogenital and Prostate (ANZUP) Cancer Trials Group, Sydney, Australia; 7 Mind Potential, Sydney, Australia; 8 School of Psychology, Griffith University, Brisbane, Australia; 9 Central Coast Cancer Centre, Gosford, Australia; 10 Concord Cancer Centre, Concord Repatriation General Hospital, Concord, Australia; 11 National Health and Medical Research Council Clinical Trials Centre, University of Sydney, Sydney, Australia; 12 Department of Social and Behavioral Sciences, Temple University, Philadelphia, PA USA; 13 Department of Surgery, Faculty of Medicine, Monash University, Melbourne, Australia; 14 Department of Urology, Monash Health, Melbourne, Australia; 15 Department of Urology, Royal Brisbane and Women’s Hospital, Brisbane, Australia; 16 Monash University, Melbourne, Australia; 17 Eastern Health, Melbourne, Australia; 18 Cancer Council NSW, Sydney, Australia

**Keywords:** Quality of life, Advanced prostate cancer, Psychological distress

## Abstract

**Objective:**

To examine the extent to which mindfulness skills influence psychological distress and health-related quality of life (HRQOL) in men with metastatic or castration-resistant biochemical progression of prostate cancer.

**Patients and methods:**

A cross-sectional survey of 190 men (46 % response; mean age 71 years, SD = 8.7, range 40–91 years) with advanced prostate cancer, assessed psychological and cancer-specific distress, HRQOL. Mindfulness skills were assessed as potential predictors of adjustment outcomes.

**Results:**

Overall, 39 % of men reported high psychological distress. One third had accessed psychological support previously although only 10 % were under current psychological care. One quarter had accessed a prostate cancer support group in the past six months.

Higher HRQOL and lower cancer-specific and global psychological distress were related to non-judging of inner experience (*p* < 0.001).

Higher HRQOL and lower psychological distress were related to acting with awareness (*p* < 0.001). Lower distress was also related to higher non-reactivity to inner experience and a lower level of observing (*p* < 0.05).

**Conclusions:**

Men with advanced prostate cancer are at risk of poor psychological outcomes. Psychological flexibility may be a promising target for interventions to improve adjustment outcomes in this patient group.

**Clinical Trial Registry:**

Trial Registration: ACTRN12612000306819

## Introduction

Prostate cancer is the second most frequently diagnosed cancer in men worldwide, with an estimated 1.1 million new cases diagnosed in 2012 [1]. It is the fifth most common cause of cancer death internationally. Incidence rates vary 25-fold worldwide, with highest rates in Australia and New Zealand. An estimated 1 in 5 Australian men will develop prostate cancer in their lifetime, and 1 in 28 will die of prostate cancer [2]. Approximately, 5–10 % of newly diagnosed Australian men have locally advanced or metastatic disease [3, 4]. Although prostate cancer is generally a slow-growing cancer, recurrence or progression can develop over the long term, even among patients considered to have low risk at diagnosis [5]. Further, an estimated 1 in 5 Australian men diagnosed with localised disease progress to metastatic disease [6].

Most prostate cancer deaths arise as a result of disease progression, and historically the median survival for men with metastatic castrate-resistant prostate cancer has been less than 2 years. The relatively recent availability of new agents for advanced disease has provided greater hope of cancer control for these men as trials indicate improved survival time [7]. However, the reality is that advanced prostate cancer is an incurable disease, and for many men, their disease progression is associated with more frequent healthcare interventions due to increasing morbidities from disease and treatments [8], diminished quality of life [9], increased psychological burden [10] and an increased risk of suicide [11, 12].

To date no randomised controlled trials have reported interventions to improve psychological outcomes for men with advanced prostate cancer, with clinicians relying on studies in men with localised disease for practice recommendations [13]. Given that the psychological challenges of advanced versus localised disease will differ (i.e. hope for cure vs. knowledge of progression), this extrapolation is likely inappropriate. In developing psychological interventions for such men, a first step is to consider what modifiable variables might be relevant therapeutic targets in a context where disease is progressive and the future highly threatened. We propose that psychological flexibility may be important for men confronting the novel, complex and uncertain challenges of advanced prostate cancer. Psychological flexibility can be broadly defined as the ability to shift mindsets in the face of changing and challenging situational demands. This includes being more aware of and able to accommodate, rather than deny or distract from, unpleasant emotions and physical morbidities and from this guide ones thoughts and actions in a constructive direction [14]. Psychological flexibility is a high level and overarching construct that includes both acceptance and mindfulness, with these processes proposed as relevant to both clinical and general populations [15]. At its centre is an interaction between psychological content, the present moment and chosen values. Mindfulness is a key and core component that speaks to the cognitive fusion and experiential avoidance that is proposed to contribute to psychological inflexibility [16]. In both laboratory and clinical settings, greater psychological flexibility has been found to be linked to more positive psychological outcomes. Finally, psychological flexibility is proposed to be stable over time, but importantly appears to be amenable to psychological intervention and as such a potential intervention target [17].

Interventions to encourage psychological flexibility, and in particular mindfulness facets or skills, are increasingly being applied in cancer populations as a psychotherapeutic approach to improving adjustment after cancer. Mindfulness approaches aim to lead the person to be less reactive to difficult experiences and to approach equanimity regarding the illness experience [18]. These approaches assume therefore a connection between these psychological facets and a person’s psychological outcomes and quality of life. However, to our knowledge no research to date has tested this assumption in men with prostate cancer, and hence it is unknown empirically whether the facets of mindfulness are related to these patients’ adjustment outcomes. Accordingly, the present study aimed to describe psychological distress and quality of life in men with advanced prostate cancer and examine the influence of mindfulness on these outcomes.

## Patients and methods

The present study utilised baseline data from an Australian randomised controlled trial of a mindfulness intervention for men with advanced prostate cancer [19]. Eligible participants were men with metastatic prostate cancer or castration-resistant biochemical progression who were referred to the trial by their treating medical specialist. Other eligibility criteria included: ability to read and speak English; no history of head injury, dementia or current psychiatric illness; and no concurrent cancer. All participants provided written informed consent. The trial received approval from the Griffith University Human Research Ethics Committee and the human research ethics committees of participating hospitals across Australia. Further detail about this trial is provided in Chambers et al. [19].

Sociodemographic information was collected in a telephone interview. Trained researchers used a data collection protocol to obtain clinical, disease and treatment information through medical records review. Psychosocial measures were completed by participants via mailed self-administered questionnaires. These measures are outlined below.

### Mindfulness facets

The 39-item Five Facet Mindfulness Questionnaire (FFMQ) measures engagement with the principles of mindfulness and contains five subscales: observing or noticing ones reaction; being able to describe this reaction; acting with awareness; non-judging of inner experience; and non-reactivity to inner experience [20]. Items were scored 1 (*never or very rarely true*) to 5 (*very often or always true*) and summed to create subscale scores with higher scores indicating greater engagement with each principle or facet (score range for observing, describing, acting with awareness, non-judging of inner experience 8–40; non-reactivity to inner experience 7–35). Internal reliability was acceptable for subscales (*α* = 0.78–0.90) and the total scale (*α* = 0.86).

### Health-related quality of life

The 39-item Functional Assessment of Cancer Therapy-Prostate (FACT-P) assesses men’s perceived global quality of life across five domains: physical, social/family, emotional, functional well-being and prostate cancer-specific concerns [21]. For this study, items were scored 0 (*not at all*) to 5 (*very much*) and averaged to create subscale scores, with these subscale scores then summed to create a global quality of life score (score range 0–156). Higher scores indicated greater perceived quality of life (*α* = 0.91). The average FACT-P total score for men with advanced disease was reported by Esper et al. as 109.8 [21].

### Psychological distress

The Brief Symptom Inventory-18 (BSI-18) provides a global measure of current psychological distress with subscale scores for anxiety, depression and somatisation [22]. In the current study, the 18 items were scored 0 (*not at all*) to 4 (*extremely*) and summed to create a Global Severity Index (GSI) with higher scores indicating greater distress (score range 0–72; *α* = 0.91). Raw scores were transformed into standardised t scores to determine the proportion of men who met the criteria for caseness. Caseness has been reported as a standardised *t* score of 57 or above on the GSI or any two subscales in men with cancer [23]. This cut-off score was used to indicate the percentage of men with clinical psychological distress in this sample.

### Cancer-specific distress

The 15-item Impact of Events Scale (IES) measures men’s cancer-specific distress and contains two subscales: intrusive symptoms and avoidance symptoms [24]. Items were scored 0 (*not at all*) to 5 (*extremely*) and summed to create an overall score with higher scores indicating greater distress (score range 0–75; *α* = 0.93). A score of 20 or above on either the intrusive or avoidance symptoms subscale was used to calculate the proportion of men with clinical cancer-specific distress, and this is in line with the cut-off score used for the IES in advanced cancer patients [25].

### Statistical analyses

Three hierarchical regressions examined the factors associated with quality of life, cancer-specific distress and psychological distress. Variables were entered into the regression in the following order: Step 1: sociodemographic and clinical characteristics (age, marital status, education level, the presence of a limiting comorbidity, time since diagnosis) and Step 2: the five facets of the FFMQ (observing, describing, acting with awareness, non-judging of inner experience, non-reactivity to inner experience). Categorical variables of marital status (1 married/de facto; 0 single), education level (1 tertiary; 0 high school or less) and the presence of a limiting comorbidity (1 yes; 0 no) were coded dichotomously for the analysis. Pairwise deletion was used for missing data.

## Results

### Patients

Between September 2012 and January 2015, 472 patients were referred to participate in the study; of these 190 completed the assessment (61 were ineligible and 221 declined to participate). Thirteen of the 190 men self-referred to the project team in response to media about the project and were assessed for eligibility prior to recruitment. The sociodemographic characteristics, self-reported health status and prostate cancer history are reported in Table 1. For prostate cancer history, medical record data were not obtainable for all patients and are reported accordingly.

**Table 1:** Sample sociodemographic characteristics, self-reported health status and prostate cancer history (*n* = 190)

Variable	
Age	70.8 years (8.7 years)
Married or de facto relationship	75 %
Retired	68 %
Born in Australia	66 %
University or college degree	66 %
*BMI*	
Overweight range	47 %
Obese range	28 %
*Smoking history*	
Ex-smoker	45 %
Current smoker	7 %
*Comorbid health conditions*	
Any condition	92 %
Back pain	59 %
Osteoarthritis or degenerative arthritis	54 %
High blood pressure	48 %
Depression or anxiety	27 %
Heart disease	22 %
Diabetes	18 %
Lung disease	11 %
At least one condition limited current activities	58 %
Time since prostate cancer diagnosis	6 years (4.9 years)
Gleason score ≥ 8^a^	71 %
*Prostate cancer stage* ^b^	
T2	20 %
T3	55 %
T4	24 %
PSA level^c^	50.6 ng/mL* (106.3 ng/mL)
*Prostate cancer treatment* ^d^	
Androgen deprivation therapy	97 %
Radiation therapy	69 %
Prostatectomy	44 %
Chemotherapy	31 %
Active surveillance	4 %
Watchful waiting	4 %
Orchidectomy	2 %

Values in parentheses are standard deviations for continuous variables

* PSA range = 0.01–588.9 ng/mL; PSA median = 7.94 ng/mL

^a^ *n* = 114; ^b^ *n* = 83; ^c^ *n* = 160; ^d^ *n* = 171

### Psychological care and support

Thirty-nine per cent of men met the criteria for clinical psychological distress as indicated by the BSI-18 [23]. Eighteen per cent of men met the criteria for clinical cancer-specific distress as indicated by the IES [25]. However, only 6 % of participants were currently receiving psychological care (psychiatrist, 2 %; psychologist, 4 %), and 11 % were taking medication for depression or anxiety. Thirty-four per cent had accessed some psychological care in the past (psychiatrist 10 %; psychologist 12 %; counsellor 14 %).

In the 6 months prior to the study, 56 % of men had received support for prostate cancer and this was predominantly from prostate cancer support groups (26 %), their doctor (26 %), books or brochures provided by their doctor (26 %), or the Internet (26 %). Men also received support from family or friends (17 %), a nurse or other health professional (16 %) and prostate cancer related newsletters (13 %). Although 58 % of participants reported previous use of the Internet for information about their prostate cancer, only 7 % used this to access online support groups or other social services.

### Quality of life

Table 2 provides the descriptive statistics and inter-correlations for all variables in the main analyses. Sociodemographic and clinical characteristics entered at Step 1 of the hierarchical regression explained 13.3 % of the variance in quality of life, *F*(5, 149) = 4.58, *p* < 0.001. Limitation by comorbidity was the only significant predictor of quality of life at this step (*B* = −13.44, SE = 3.09, *β* = −.33, *p* < 0.001). The addition of the five facets of mindfulness in Step 2 significantly increased the explained variance by 30.4 %, *F*(5, 144) = 15.52, *p* < 0.001. Limitation by comorbidity remained a significant predictor of quality of life at this step contributing 2.8 % unique variance. Of the five facets acting with awareness and non-judging of inner experience were the only significant predictors of quality of life, contributing 3.1 and 9.3 % unique variance, respectively. Both of these facets had a positive relationship with quality of life (Table 3).

**Table 2:** Descriptive statistics and inter-correlations among analysis variables

Variable	M (SD)	1	2	3	4	5	6	7	8	9	10	11	12	13
1. QOL	111.74 (20.12)													
2. Cancer-specific distress	15.72 (15.58)	−.59**												
3. Psychological Distress	52.40 (10.32)	−.73**	.64**											
4. Age	70.79 (8.68)	.11	−.15*	−.14										
5. Marital status^a^		.04	.01	−.05	−.05									
6. Education^a^		.05	.09	−.04	−.15	−.04								
7. Limited by comorbidity^a^		−.35**	.27**	.30**	−.06	.12	−.09							
8. Time since diagnosis	5.95 (4.89)	.14	−.15	−.07	.24*	.05	−.06	.03						
9. Observing	22.33 (6.73)	−.05	.15*	.18*	−.01	.19*	.11	.25*	−.07					
10. Describing	28.54 (6.63)	.30**	−.27**	−.31**	.06	.06	.15	−.03	.02	.28**				
11. Acting with awareness	31.39 (6.10)	.51**	−.51**	−.56**	.12	.05	.04	−.30**	.05	−.14	.42**			
12. Non-judging of inner experience	31.93 (7.00)	.53**	−.60**	−.57**	.00	.04	.07	−.24*	.04	−.27**	.25**	.55**		
13. Non-reactivity to inner experience	20.78 (5.82)	.06	.05	−.03	−.03	.04	.12	.08	−.01	.53**	.37**	−.04	−.11	

Range of obtained scores for QOL 39–151; cancer-specific distress 0–61; psychological distress 0–45; age 40.38–91.80 years; time since diagnosis 0–25.90 years; observing 8–38; describing 11–40; acting with awareness 15–40; non-judging of inner experience 12–40; non-reactivity to inner experience 7–34

* *p* < 0.05; ** *p* < 0.001

^a^Categorical variable; correlations calculated using Spearman’s Rho

**Table 3:** Final step of hierarchical regression predicting quality of life (*n* = 155)

Predictors	*B*	SE	*β*
*Sociodemographic and clinical characteristics*			
Age	−.03	.15	−.01
Marital status	.37	3.00	.01
Education	−.83	2.73	−.02
Limited by a comorbidity	−7.27	2.74	−.18*
Time since diagnosis	.37	.26	.09
*Mindfulness facets*			
Observing	.09	.26	.03
Describing	.16	.24	.05
Acting with awareness	.77	.27	.23*
Non-judging of inner experience	1.10	.23	.39**
Non-reactivity to inner experience	.36	.27	.11

At the final step, the overall model was significant and explained 43.7 % of the variance in quality of life, *F*(10, 144) = 11.17, *p* < 0.001

* *p* < 0.05; ** *p* < 0.001

### Cancer-specific distress

In Step 1, sociodemographic and clinical characteristics explained 8.2 % of the variance in cancer-specific distress, *F*(5, 148) = 2.66, *p* = 0.02. Limitation by comorbidity was the only significant predictor of cancer-specific distress at this step (*B* = 7.14, SE = 2.59, *β* = .22, *p* < 0.01). The addition of the five facets of mindfulness in Step 2 significantly increased the explained variance by 42.8 %, *F*(4, 143) = 24.98, *p* < 0.001. At this final step, non-judging of inner experience was the only significant predictor of cancer-specific distress and contributed 18.3 % of unique variance. Greater non-judging was related to lower cancer-specific distress. Limitation by comorbidity was no longer significant at the second step with the addition of the mindfulness facets to the model (Table 4).

**Table 4:** Final step of hierarchical regression predicting cancer-specific distress (*n* = 154)

Predictors	*B*	SE	*β*
*Sociodemographic and clinical characteristics*			
Age	−.12	.12	−.06
Marital status	.73	2.30	.02
Education	3.57	2.07	.11
Limited by a comorbidity	.81	2.08	.02
Time since diagnosis	−.20	.20	−.06
*Mindfulness facets*			
Observing	.05	.19	.02
Describing	−.21	.18	−.09
Acting with awareness	−.39	.21	−.15^a^
Non-judging of inner experience	−1.25	.17	−.55**
Non-reactivity to inner experience	−.03	.21	−.01

At the final step, the overall model was significant and explained 51.0 % of the variance in cancer-specific distress, *F*(10, 143) = 14.89, *p* < 0.001

* *p* < 0.05; ** *p* < 0.001; ^a^ *p* = 0.057

### Psychological distress

Sociodemographic and clinical characteristics in Step 1 explained 10.2 % of the variance in psychological distress, *F*(5, 148) = 3.36, *p* < 0.01. Limitation by comorbidity was the only significant predictor of psychological distress at this step (*B* = 6.11, SE = 1.65, *β* = .29, *p* < 0.001). In Step 2, the addition of the five facets of mindfulness significantly increased the explained variance by 41.3 %, *F*(5, 143) = 24.33, *p* < 0.001. Four out of the five facets were significant predictors of psychological distress at this final step. Non-judging of inner experience contributed the most unique variance (10 %) followed by acting with awareness (4.5 %), non-reactivity to inner experience (3.2 %) and observing (1.6 %). Each facet had a negative relationship with psychological distress with the exception of observing which had a positive relationship with distress. Limitation by comorbidity was no longer significant at the second step with the addition of the mindfulness facets to the model (Table 5).

**Table 5:** Final step of hierarchical regression predicting psychological distress (*n* = 154)

Predictors	*B*	SE	*β*
*Sociodemographic and clinical characteristics*			
Age	−.06	.08	−.05
Marital status	−1.08	1.46	−.04
Education	.23	1.33	.01
Limited by a comorbidity	1.88	1.33	.09
Time since diagnosis	−.01	.13	−.00
*Mindfulness facets*			
Observing	.27	.12	.17*
Describing	−.12	.11	−.08
Acting with awareness	−.48	.13	−.28**
Non-judging of inner experience	−.60	.11	−.40**
Non-reactivity to inner experience	−.41	.13	−.23*

At the final step, the overall model was significant and explained 51.5 % of the variance in psychological distress, *F*(10, 143) = 15.16, *p* < 0.001

* *p* < 0.05; ** *p* < 0.001

## Discussion

Many of the men in this study reported high levels of psychological distress, with health-related quality of life similar to previous research with men with advanced prostate cancer [21]. Importantly, three key psychological mechanisms or mindfulness facets were associated with better outcomes: awareness, non-judgement and non-reactivity. Evidence suggests that people who judge their (especially negative) experiences can end up in a ruminative loop of “why am I feeling this way” which ironically has the effect of worsening distress [26]. Our results support this contention and suggest that interventions that promote awareness paired with non-judgement and non-reactivity may be useful in the setting of advanced prostate cancer. With regard to intervention research, to date a few preliminary studies have reported applying mindfulness approaches with men with prostate cancer. One single-arm trial of mindfulness-based stress reduction groups with 49 breast cancer patients and 10 men with localised prostate cancer found post-intervention improvements in quality of life and stress symptoms [27], with benefits maintained over time [28]. In a more recent study, men with advanced prostate cancer who participated in a mindfulness-based cognitive intervention targeting self-awareness, non-judgement and acceptance, reported moderate to large improvements in anxiety and fear of cancer recurrence [29]. In the present study, non-judging of inner experience demonstrated the strongest effect across both quality of life and psychological distress and this may be of particular relevance given masculine values around stoicism in the face of adversity that may exacerbate distress and isolation in a chronic illness [30]. Specifically, as cancer progresses and fears and concerns about the future naturally arise, a stoic approach may become difficult to maintain. Hence the development of a less judgemental and more flexible approach to coping may be crucial for men facing advanced prostate cancer. These are important and novel findings.

Despite high distress, most men were currently not receiving psychological care. This is consistent with previous research showing that men with prostate cancer often report unmet psychological needs [31] and again reinforces the need for action to detect men with high distress and provide targeted intervention [32]. Taking into account the apparent strong role of mindfulness facets across both QOL and distress, acceptance focussed approaches such as mindfulness-based cognitive therapies may be indicated [33]. Uptake of support groups was high in this cohort and was more accessed than professional psychological care. This may relate at least in part to the social isolation that can be experienced when cancer has advanced leading a person to seek support by connecting with others in a similar situation [34, 35]. These approaches appear worthy of future research.

It is noteworthy that being limited by comorbid conditions was an independent predictor of HRQOL, but not cancer-specific or global distress. This may be due to the fact that the HRQOL measure captures limitations in social and physical functioning which might be directly due to comorbid conditions, particularly arthritis and back pain that were highly prevalent in this population. The finding that physical limitations imposed by comorbid conditions were not strong predictors of distress after psychological flexibility was entered into the models suggests that this flexibility may mediate the distressing effects of other physical health problems, not just prostate cancer.

Limitations of the present study include the cross-sectional design such that causality cannot be inferred. However, the inclusion of modifiable psychological variables using well-validated assessment measures is novel, and the use of a relatively large national sample is a strength. Future longitudinal research is needed to test if these relationships persist over time. As well, the participation rate was only 46 % with men in the study reporting high levels of education. It may be that men who did not participate differed in other background or clinical characteristics as well as their levels of distress and as a result our study may well underrepresent levels of distress in this vulnerable patient group. Finally, we note that the observed facet was associated with greater distress, a direction of effect consistent with earlier research suggesting this subscale may not capture the quality of noticing one’s experience that is central to mindfulness-based approaches [20]. Future research in the cancer context is needed to expand assessments of psychological flexibility beyond mindfulness to include other processes, such as values exploration and committed action and from this fine-tune potential therapy targets [16].

In conclusion, several facets of mindfulness may hold promise as therapeutic targets to reduce psychological distress and improve QOL in men with advanced prostate cancer. Future longitudinal descriptive research is needed to examine the influence of not only psychological flexibility in its broader definition, but also other relevant constructs such as masculinity [36]. This would assist both practitioners and researchers to better understand the dynamic of how men adjust and learn to live with serious and chronic illness. Finally, larger randomised controlled trials controlled trials are needed to move knowledge forward in the effectiveness of psychological interventions for men with cancer.
